# Complete Genome Sequence of Bacillus megaterium Strain TG1-E1, a Plant Drought Tolerance-Enhancing Bacterium

**DOI:** 10.1128/MRA.00842-18

**Published:** 2018-09-27

**Authors:** Juan Ignacio Vílchez, Qiming Tang, Richa Kaushal, Wei Wang, Suhui Lv, Danxia He, Zhaoqing Chu, Heng Zhang, Renyi Liu, Huiming Zhang

**Affiliations:** a Shanghai Center for Plant Stress Biology and CAS Center for Excellence in Molecular Plant Sciences, Chinese Academy of Sciences, Shanghai, China; b Shanghai Key Laboratory of Plant Functional Genomics and Resources, Shanghai Chenshan Plant Science Research Center, Shanghai, China; c University of Chinese Academy of Sciences, Beijing, China; d Center for Agroforestry Mega Data Science and FAFU-UCR Joint Center for Horticultural Biology and Metabolomics, Haixia Institute of Science and Technology, Fujian Agriculture and Forestry University, Fuzhou, China; University of Arizona

## Abstract

Based on a combination of next-generation sequencing and single-molecule sequencing, we obtained the whole-genome sequence of Bacillus megaterium strain TG1-E1, which is a highly salt-tolerant rhizobacterium that enhances plant tolerance to drought stress. The complete genome is estimated to be approximately 5.48 Mb containing a total of 5,858 predicted protein-coding DNA sequences.

## ANNOUNCEMENT

Our group characterized Bacillus megaterium strain TG1-E1 as a highly salt-tolerant Gram-positive bacterium that is capable of enhancing plant tolerance to drought stress. It was originally isolated from a rhizospheric soil sample of Spartina anglica at Zhangpu Yanchang in Fujian Province, China. This rhizobacterium collection is rich in specimens of the *Firmicutes* and *Proteobacteria* phyla, with about 70% belonging to the *Bacillaceae* family. High salinity in the sampling area possibly contributes to the enrichment of *Bacillus* strains in the rhizosphere (1–4). In addition, more than half of the strains isolated in this sampling area can produce phytometabolites, such as auxins and aminocyclopropane-1-carboxylate deaminase (ACCd), displaying the characteristics commonly described in plant tolerance-enhancing strains (5–10). B. megaterium TG1-E1 has been deposited in the China General Microbiological Culture Collection Center (CGMCC) with reference number 14422.

DNA samples (at least 100 nM in 10 µl) were obtained from bacteria grown in LB medium until an optical density of 1 at 600 nm (OD_600_) was obtained. The sequencing of the B. megaterium TG1-E1 genome was completed by combining next-generation sequencing (NGS) and single-molecule sequencing. NGS was performed with 20 µg of DNA with an Illumina HiSeq platform (Core Facility of Genomics, Shanghai Center for Plant Stress Biology, China), and single-molecule sequencing was performed with 20 µg of DNA with a PacBio platform (Tianjin Biochip Corporation, China) (11–14). The shotgun sequencing strategy was applied to NGS, and 12,471,203 paired reads (150 bp) were obtained with a sequencing depth of approximately 260-fold. Meanwhile, single-molecule sequencing produced 98,959 reads with a mean read length of 10,551 bp and an *N*_50_ length of 14,471 bp. The total number of sequenced bases was 961,774,920. For *de novo* assembly, Canu v1.5 was used with default parameters, and the genome correction step was performed using Illumina data with support of Pilon v1.18 (15, 16). The size of the circularized genome was calculated to be about 5.48 Mb. Genes including protein-coding DNA sequences (CDSs) were predicted by a pipeline implemented by Prokka v1.12 (17). On a whole-genome scale, The GC content of this genome is 38.26%, and 5,858 protein-coding genes, 4 rRNA operons, and 164 tRNA genes were called during annotation.

The whole-genome sequence of B. megaterium TG1-E1 reveals information such as the biosynthesis pathways of flagella, spores, and polysaccharides. Concerning characteristics potentially contributing to TG1-E1-induced plant stress tolerance, pathways found within this genome that have potential relevance in aiding plant drought stress include trehalose and antioxidant biosynthesis. In addition, genome annotation also revealed possible mechanisms for plant growth-promoting effects, including bacterial production of acid phosphatases, siderophores, and exopolysaccharides. Further research with this genomic information will help us discover mechanisms through which B. megaterium TG1-E1 induces plant drought stress tolerance and will contribute to the subsequent development of biotechnological applications.

### Data availability.

The complete genome sequence of B. megaterium TG1-E1 has been deposited in the TBL/EMBL/GenBank databases under the BioProject number PRJNA430758 and the accession number PRKV00000000 (sequences PRKV01000001 to PRKV01000036).
